# Comparison of simulation and predictive efficacy for hemorrhagic fever with renal syndrome incidence in mainland China based on five time series models

**DOI:** 10.3389/fpubh.2024.1365942

**Published:** 2024-02-29

**Authors:** ZhenDe Wang, ChunXiao Yang, Bing Li, HongTao Wu, Zhen Xu, ZiJian Feng

**Affiliations:** ^1^School of Public Health, Shandong Second Medical University, Weifang, China; ^2^Chinese Center for Disease Control and Prevention, Beijing, China; ^3^National Key Laboratory of Intelligent Tracking and Forecasting for Infectious Diseases, Beijing, China; ^4^Chinese Preventive Medicine Association, Beijing, China

**Keywords:** HFRS, ARIMA, LSTM, CNN, NARX

## Abstract

**Background:**

Hemorrhagic fever with renal syndrome (HFRS) is a zoonotic infectious disease commonly found in Asia and Europe, characterized by fever, hemorrhage, shock, and renal failure. China is the most severely affected region, necessitating an analysis of the temporal incidence patterns in the country.

**Methods:**

We employed Autoregressive Integrated Moving Average (ARIMA), Long Short-Term Memory (LSTM), Convolutional Neural Network (CNN), Nonlinear AutoRegressive with eXogenous inputs (NARX), and a hybrid CNN-LSTM model to model and forecast time series data spanning from January 2009 to November 2023 in the mainland China. By comparing the simulated performance of these models on training and testing sets, we determined the most suitable model.

**Results:**

Overall, the CNN-LSTM model demonstrated optimal fitting performance (with Root Mean Square Error (RMSE), Mean Absolute Percentage Error (MAPE), and Mean Absolute Error (MAE) of 93.77/270.66, 7.59%/38.96%, and 64.37/189.73 for the training and testing sets, respectively, lower than those of individual CNN or LSTM models).

**Conclusion:**

The hybrid CNN-LSTM model seamlessly integrates CNN’s data feature extraction and LSTM’s recurrent prediction capabilities, rendering it theoretically applicable for simulating diverse distributed time series data. We recommend that the CNN-LSTM model be considered as a valuable time series analysis tool for disease prediction by policy-makers.

## 1 Introduction

Hemorrhagic Fever with Renal Syndrome (HFRS) is a zoonotic infectious disease caused by Hantavirus infection (1). Human hantavirus infection can lead to two clinical syndromes: hemorrhagic fever with renal syndrome (HFRS) and hantavirus cardiopulmonary syndrome (HCPS) (2), the former mainly prevalent in Europe and Asia, and the latter mainly distributed in the Americas (3). The pathogenic hantavirus is carried by specific rodent hosts in the natural environment, and the virus spreads with the excrement of the animals. Infection caused by inhaling dust or aerosols containing these viruses is the main mode of transmission for this virus (4). HFRS is typically characterized by five phases: febrile, hypotensive, oliguric, diuretic, and convalescent, usually following a latent period of 2–3 weeks. Acute kidney injury is a hallmark of HFRS, manifesting as renal enlargement, proteinuria, and hematuria (5). The clinical symptoms of HFRS include fever, headache, back pain, visual disturbances, gastrointestinal symptoms (such as nausea, vomiting, diarrhea, and melena), and proteinuria (1). Disseminated intravascular coagulation (DIC) may also occur in severe cases of HFRS. Although complete renal recovery can occur after a prolonged convalescent period, chronic renal failure and hypertension may also develop. In addition to typical renal manifestations of HFRS, some patients may also experience extrarenal symptoms, such as acute respiratory distress syndrome (ARDS), cholecystitis, pericarditis, and encephalitis (5, 6).

Although hantavirus was first reported in the 1980s, the diseases it causes have been recorded for much longer (7, 8). Currently, over 28 pathogenic species of hantavirus are known, and there are still many potential species yet to be discovered. Globally, about 150,000 to 200,000 HFRS or HCPS/HPS cases are reported annually, with case fatality rates (CFR) ranging from 1 to 15% for HFRS and 30–50% for HCPS/HPS (6). Between 1978 and 1995, 15 out of 29 regions in Asian Russia reported a total of 3,145 HFRS cases (8). During 2000–2022, a total of 69 of Russia’s 85 administrative regions reported 164,580 HFRS cases, with an annual average rate of 4.9 cases/100,000 population. European Russia reported 162,045 (98.5%) cases in 53/60 regions with 9.7 cases/105 population. Asian Russia reported 2,535 (1.5%) cases in 16/25 regions with 0.6 cases/105 population (9). During the Korean War (1950–1953), approximately 1,700 American soldiers were found to have symptoms consistent with HFRS, with less than 5% resulting in fatalities. Later, symptoms similar to the Far Eastern HFRS were identified in patients in the Scandinavian Peninsula (10). China has the most severe outbreak of HFRS, accounting for nearly 90% of all HFRS cases (6, 11, 12), and has been widely studied and monitored by public health scholars and officials. Reported cases in China increased from 10,378 between 1931 and 1949 (13) to 1,557,622 between 1950 and 2007 (14). Despite this, China remains the most severely affected area by HFRS, with a total of 209,209 cases registered between 2004 and 2019, including 1,855 fatalities (15). Shandong Province, Heilongjiang Province, Hunan Province, Jiangxi Province, and Zhejiang Province have been identified as hotspots for HFRS incidence (16). Increased vascular permeability is a core manifestation of the pathogenesis of HFRS, but researchers have found that the mechanism of HFRS is complex, causing systemic damage, and there is currently no specific treatment (1). Therefore, public health intervention is the most prospective and effective measure to reduce its transmission risk and harm (9).

To appropriately prepare for potential peaks in disease incidence, policy-makers must preemptively devise public health interventions and emergency plans that align with the observed trends and epidemiological patterns of diseases. Time series data of disease incidence serve as a fundamental resource for reflecting trends in disease epidemiology (17). Such data not only provide a visual representation of long-term trends in disease prevalence but also encapsulate additional information pertinent to the unique characteristics of disease outbreaks. Consequently, the analysis and modeling of incidence rate time series have become prevalent research methodologies.

Current time series modeling techniques predominantly include Autoregressive Integrated Moving Average (ARIMA) models, Generalized Autoregressive Conditional Heteroskedasticity (GARCH) models, and various machine learning models. Researchers frequently employ these time series models for constructing predictive frameworks, which often demonstrate significant efficacy in simulation and forecasting applications (18–21). The ARIMA model is suitable for modeling stationary time series data and is the most widely used traditional time series model. The model is a valuable tool for analyzing stationary time series data with seasonal fluctuations. However, its ability to capture nonlinear components within the data is limited. Furthermore, the application of the ARIMA model is contingent upon stringent conditions, often necessitating the use of differencing operations or logarithmic transformations. These procedures can result in a loss of information from the original dataset. Additionally, an excessive number of differencing operations can reduce the efficiency of utilizing the information contained in the original data, as evidenced by the probability distribution and the autoregressive effects of the model residuals. In recent years, with the rapid development of machine learning technology, an increasing number of algorithms have been applied in the field of time series analysis. Among these, Artificial Neural Networks (ANNs), as an important algorithm in machine learning, were initially inspired by the biological structure of human neurons (22). They have been widely applied by researchers to construct various types of time series models across different domains. Unlike feed-forward or feed-back neural networks, Recurrent Neural Networks (RNNs) rely not only on the current input but also on the previous output at a given moment (23). Therefore, RNNs are less prone to the vanishing or exploding gradient problem in the analysis of sequence data with longer time spans. Within RNNs, Long Short-Term Memory (LSTM) and Nonlinear Autoregressive Exogenous (NARX) neural networks with external inputs are the two most typical neural network models. LSTM, through its gating mechanism and memory unit, can capture long-term dependencies in sequence data and perform contextual modeling, thus demonstrating outstanding performance in modeling and predicting sequence data (24). Although the LSTM model mitigates the issue of gradient vanishing or exploding during training through its unique gating units, it does not entirely eliminate the problem (25). Additionally, RNN models, including LSTM and NARX, are characterized by computational complexity, lengthy training times, and sensitivity to hyperparameters. Therefore, conducting experiments with multiple parameter combinations is necessary to determine the optimal set, which inevitably increases time costs. Convolutional Neural Networks (CNNs) are a widely used tool in the field of deep learning, with particular strength in processing image inputs (26, 27). However, their applicability extends to other domains such as text, signals, and other forms of continuous responses (28). A CNN is composed of multiple layers, including convolutional layers, pooling layers, and fully connected layers, it employs an array of convolutional filters to perform feature extraction from raw input, capitalizing on its intrinsic property of spatial locality recognition. Through the mechanism of weight sharing across convolutional layers, the CNN efficiently compresses the computational demand while simultaneously ensuring the thorough assimilation of information inherent in the input data. Nonetheless, the essence of the CNN as a feed-forward neural network introduces a backpropagation paradigm for weight updates (29), which can result in a protracted rate of adjustment for weights proximal to the input layer. Additionally, the employment of gradient descent optimization algorithms predisposes the training outcomes to convergence upon local optima, rather than the global optimum, posing a significant challenge in the network’s learning process. Considering the CNN model’s proficient capability in extracting features from raw data and the LSTM’s remarkable long-term memory ability, we have integrated the CNN and LSTM models to construct a hybrid CNN-LSTM model for the purpose of fitting and forecasting. In this study, ARIMA, LSTM, NARX, CNN, and CNN-LSTM models were selected to model the monthly incidence time series of HFRS in mainland China from January 2009 to October 2023. The study compared the goodness of fit of these models to provide clues for exploring more suitable time series modeling tools, which will be beneficial for public health policy makers to formulate more scientifically informed public health measures against potential HFRS outbreaks.

## 2 Methods

### 2.1 Data collection

Data pertaining to the monthly incidence of HFRS spanning from January 2009 to October 2023 were retrieved from the official portal of the National Health Commission’s Bureau for Disease Control and Prevention of China.^1^ This dataset was meticulously collated from the comprehensive monthly bulletins of statutorily reportable infectious maladies (30). The instantiation of case data for these reportable infectious diseases was systematically transmitted from an extensive network of local hospitals and community health service facilities distributed nationwide. Subsequent to initial reporting, each case was subjected to rigorous verification and validation processes by the respective local Centers for Disease Control and Prevention (CDC), incorporating confirmatory diagnostic evaluations. The analytical scope of this investigation encompasses a corpus of 178 discrete temporal data points.

### 2.2 Time series decomposition

In time series trend analysis, the Mann-Kendall (M-K) test is a widely used non-parametric test method for analyzing trend changes in a time series. The M-K test does not require the sample to follow a certain distribution, is not affected by a few outliers (31). In a two-sided trend test with a specified test level of *α* = 0.05, the presence of a significant increasing or decreasing trend in the sequence can be inferred if the value of |*z*| > 1.96. A *z*-value greater than *zero* signifies an upward trend, while a *z*-value less than *zero* indicates a downward trend. Time series decomposition is a technique used to break down a time series into its underlying components. The decomposition of time series not only facilitates the capture of long-term trends and seasonal attributes inherent within the data but also constitutes a prerequisite for the selection of the most appropriate ARIMA model for subsequent analysis. It helps in understanding and analyzing the individual components of a series by separating it into a deterministic and nonseasonal secular trend component (*T_t_*), a deterministic seasonal component (*S_t_*), and a stochastic irregular component (*I_t_*). The additive decomposition is appropriate to use when there is no exponential growth in the series, and the amplitude of the seasonal component remains constant over time, which is expressed as *Y_t_ = T_t_ + S_t_ + I_t_*. As the sample data is monthly, we confirmed the *T_t_* by using a smooth weighted 13-term moving average filter given by:


$$T_{t}=\overset{q}{\underset{j=-q}{\sum }}k_{j}y_{t+j}$$

Where *q = 6* for monthly data*, q < t < N-q, k_j_ = 1/4q* for *j = ±q,* and *k_j_ = 1/2q* otherwise. After the transformation of time series, the first and last six observations were lost, so we repeated the first and last smoothed values six times.

Define *n_t_* as the aggregate of observations recorded within the temporal interval (*t*). The stable seasonal filter is delineated as follows:


$$\tilde{s_{t}}=\frac{1}{n_{t}}\overset{\left(\frac{N}{s}\right)-1}{\underset{j=1}{\sum }}x_{t+js}$$

$$\bar{s}=\frac{1}{S}\;\overset{s}{\underset{t=1}{\sum }}\tilde{s_{t}}$$

$$\hat{S_{t}}=\tilde{s_{t}}-\bar{s}$$

For *s* = 12, *t*∈[1, *s*]. Using $\hat{s_{t}}$ to constrain the seasonality component to fluctuate around *zero*.

### 2.3 Modeling of ARIMA

#### 2.3.1 Mathematical equations of the ARIMA model

After an initial analysis of the time series, it was observed that the reported HFRS incidence demonstrates a cyclical oscillation with a periodicity of 12 months (32). As a result, the ARIMA(*p*, *d*, *q*)(*P*, *D*, *Q*)_12_ model is considered more appropriate for the time series analysis. Here, *p*, *d*, and *q* represent the non-seasonal components of the model, specifically the autoregression order, the differencing lag, and the moving average order, respectively. Similarly, *P*, *D*, and *Q* correspond to the seasonal components of the model, with *P* indicating the seasonal autoregression lag, *D* representing the seasonal differencing lag, and *Q* specifying the lag order of the seasonal moving average process. The polynomial expression of the ARIMA(*p*, *d*, *q*)(*P*, *D*, *Q*)_12_ model can be formulated as


$$\varphi \left(L\right)\;\Phi \left(L\right)\;\Delta^{d}\Delta_{12}^{D}\;y_{t}=\theta \left(L\right)\;\Theta \left(L\right)\;\varepsilon_{t}$$

$$L^{i}y_{t}=y_{t-i}$$

$$\Delta^{d}={\left(1-L\right)}^{d}$$

$$\Delta_{12}=\left(1-L^{12}\right)$$

$$\varphi \left(L\right)=1-\varphi_{1}L-\ldots -\varphi_{p}L^{p}$$

$$\Phi \left(L\right)=1-\Phi_{12}L-\ldots -\phi_{12P}L^{12P}$$

$$\theta \left(L\right)=1+\theta_{1}L+\ldots +\theta_{q}L^{q}$$

$$\Theta \left(L\right)=1+\Theta_{12}L+\ldots +\Theta_{12Q}L^{12Q}$$

Where $\varepsilon_{t}$ denotes a sequence of uncorrelated random variables from a defined probability distribution with a mean *zero*. φ,Φ,θ,andΘ represent the parameters to be estimated.

#### 2.3.2 ARIMA model selection and residuals diagnosis

The premise for establishing an ARIMA model is that the data must be a stationary non-white noise sequence (33). Therefore, prior to modeling, it is necessary to conduct a diagnostic test for stationarity. The Augmented Dickey-Fuller (ADF) test is a commonly used method for testing stationarity (The null hypothesis for this test is that the first *m* autocorrelations are jointly *zero*). Common methods for handling non-stationary data include differencing and logarithmic transformation. We perform first-lag differencing and seasonal differencing on the data, followed by an ADF test on the transformed series. If the series is stationary, we proceed with the Ljung-Box Q-test. If the test result rejects the hypothesis that the series is white noise, we can continue with modeling. At this point, the values of the parameters *d* and *D* in the ARIMA model indicate the number of differencing steps required to achieve stationarity. In determining the order of other model parameters, we rely on the Akaike Information Criterion (AIC) and the Bayesian Information Criterion (BIC). Information criteria are likelihood-based measures of model fit that include a penalty for complexity (specifically, the number of parameters). Different information criteria are distinguished by the form of the penalty, and can prefer different models. Let *logL*( $\hat{\theta }$ ) denote the value of the maximized loglikelihood objective function for a model with *k* parameters fit to *N* data points. The AIC and BIC for a specific model are given by the formulas: −2 *logL*( $\hat{\theta }$ ) + 2 *k,* and − 2 *logL*( $\hat{\theta }$ ) + *klog*(*N*), respectively, The AIC compares models from the perspective of information entropy, as measured by Kullback–Leibler divergence. The BIC compares models from the perspective of decision theory, as measured by expected loss (34). In comparing AIC and BIC values among multiple models, lower criterion values are preferred. We set the ranges for *p*, *q*, *P*, and *Q* as [0, 3], and for each parameter combination, and calculate the AIC and BIC values. The model with the lowest sum of AIC and BIC is considered the best-fitting model, the parameters of the model were estimated by the maximum likelihood approach. After modeling, a residuals diagnosis was needed, the residuals are smooth white noise sequences if modeling succeeds. We conducted the Ljung-Box Q*-*test, along with the autocorrelation function (ACF) and partial autocorrelation functions (PACF) plots on the residual series to check the autocorrelation. Besides, we performed normality diagnostics by plotting the histogram of standard residuals and the Quantile-Quantile (QQ) plot of residuals. We split the data into a training set (first 154 observations of series) and a test set (last 24 observations of series) and used the training set for modeling, a 24-step forward prediction was then performed using the determinated ARIMA model. Finally, the simulation performance of the training set and the test set were calculated separately.

### 2.4 Constructing the LSTM model

#### 2.4.1 Cell structure of LSTM network

**Figure 1:**
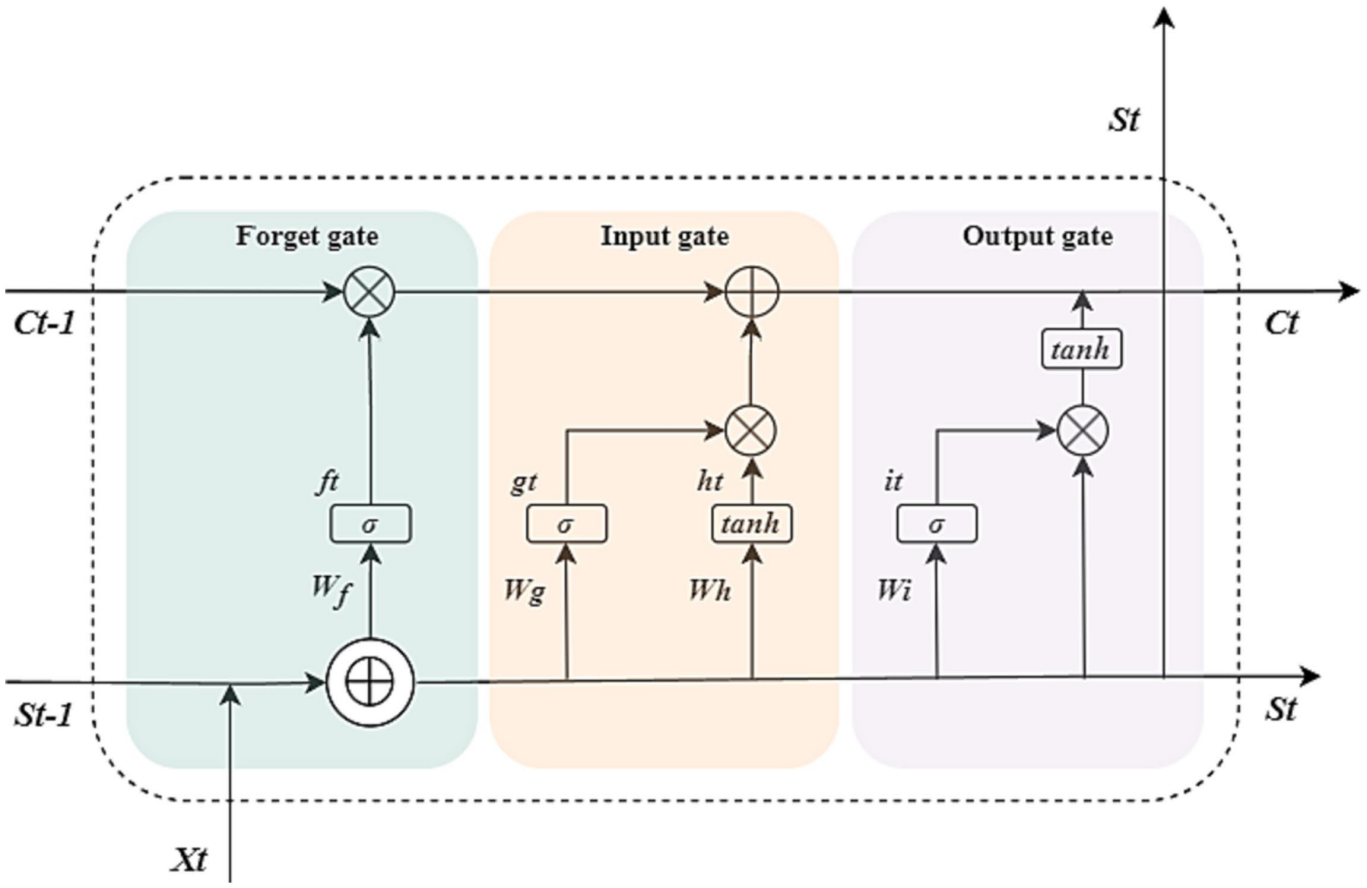
Cell structure of the LSTM network. The arrow indicates the data flow, where *x*, *s*, *f*, *g*, *h*, and *i* denote the input, output, forget gate, input gate, cell candidate, and output gate in time step *t*, respectively. *σ* and *tanh* denote the *sigmoid* activation function and the hyperbolic tangent function, which maps the data to (0, 1) and (−1, 1), respectively. ⊗ and ⊕ are vector operators which represent element-wise multiplication and element-wise addition, respectively.

The LSTM neural network model we designed consists of an input layer, LSTM layer, RELU activation layer, fully connected layer, and regression layer. The sequence input layer serves as the initial data ingress point for the neural architecture, facilitating the introduction of sequential data into the computational graph. Subsequently, the LSTM layer is tasked with discerning and preserving long-range temporal dependencies within the time step continuum of the input sequence. Distinctive from conventional RNNs, the LSTM layer incorporates a dedicated cell state mechanism, which is adept at maintaining and propagating salient long-term informational cues gleaned from antecedent temporal intervals, thereby ameliorating the issue of vanishing gradients that commonly plagues standard RNNs. At each discrete temporal juncture, the LSTM layer modulates the cell state through a series of operations—information is selectively augmented or excised based on the cell’s current context. These crucial state transitions are meticulously orchestrated by a set of adaptive gating units, which include the input, forget, and output gates, each performing a specific regulatory function to ensure the fidelity of information flow across the temporal expanse of the sequence. There are three kinds of gates in the LSTM layer, input gate, forget gate, and output gate (35). Figure 1 illustrates the flow of data at time step *t* and shows how the gates forget, update, and output the cell and hidden states.

The following formulas describe the operation of the data in the LSTM layers at time step *t.*


$$f_{t}=\sigma \left(W_{f}\cdot \left[S_{t-1},X_{t}\;\right]+b_{f}\right)$$

$$g_{t}=\sigma \left(W_{g}\cdot \left[S_{t-1},X_{t}\;\right]+b_{g}\right)$$

$$h_{t}=\tanh\left(W_{h}\cdot \left[S_{t-1},X_{t}\;\right]+b_{h}\right)$$

$$i_{t}=\sigma \left(\;W_{i}\cdot \left[S_{t-1},X_{t}\;\right]+b_{i}\right)$$

$$C_{t}=f_{t}⊗C_{t-1}+g_{t}⊗h_{t}$$

$$S_{t}=i_{t}⊗\tanh\left(C_{t}\right)$$

Where *W*, *b* denote the matrices of input weight and bias, respectively.

#### 2.4.2 Selecting the optimal LSTM model

The LSTM model does not require the data distribution to be specified, so there is no need to difference the data as in building the ARIMA model. However, in order to improve training speed and accelerate model convergence, it is necessary to normalize the data using the formula x∗=(x−min)/(max−min) to map the data range to [0, 1]. We first rearrange the time series data according to the principle of using every 5 values as input and the next value as output. For example, the first 1–5 numbers are used as input and the 6th number is used as output, the 2nd-6th numbers are used as input and the 7th number is used as output, and so on. After the transformation, we obtained a matrix with a height of 173 and a width of 6, which means that the initial 1–5 original data cannot be fitted. We use the first 1–149 rows of data as the training set and the 150–173 rows as the test set. The quantity of hidden layers within the LSTM network architecture, the maximal number of training epochs, and the initial learning rate parameter influence the outcomes of the computational simulations. Therefore, in order to select the optimal model, we fix the iteration times and learning rate as constants, and conduct multiple trainings by trying different numbers of hidden units, and then make 24-step predictions, choosing the best LSTM model based on the RMSE value of the test set. To prevent the gradients from exploding, we set the gradient threshold of the network to *one*. We used the Adaptive moment estimation(*Adam*) solver to update the network parameters by taking small steps in the direction of the negative gradient of the loss function. The solvers update the parameters using a subset of the data at each step.

We set the initial learning rate to 0.005, which is the median of the recommended range (36), and the number of maximum iterations to 1,000. We initially attempted modeling with 4 hidden units, but the performance on the test set was unsatisfactory. Therefore, we increased the number of neurons to 8, yielding similar results. Subsequently, we systematically varied the number of neurons from 4 to 150 in increments of 4, and established LSTM models, calculating the simulation performance on both the training and test sets. We also mitigated the risk of overfitting by adding regularization and implementing a phased learning rate schedule. To automatically drop the learning rate during training, using a piecewise learn rate schedule, multiply the initial learning rate by a drop factor of 0.2 after half of the maximum epochs.

### 2.5 Constructing the CNN model

**Figure 2:**
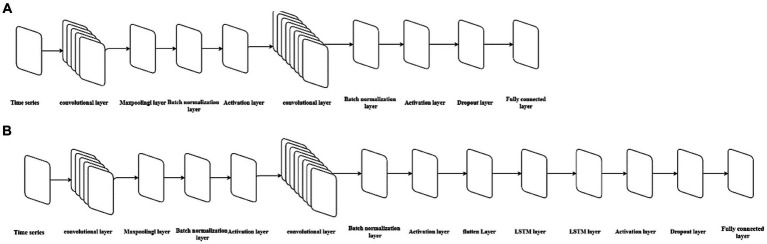
The neural architecture of the CNN model and CNN-LSTM model. **(A)** Represents the structure of the CNN model, while **(B)** represents the structure of the CNN-LSTM model.

The main feature of the CNN model is that its structure includes one or more convolutional layers. The convolutional layer consists of neurons connected to subregions of the input data or to the output of the previous layer (37). The convolutional layer contains multiple convolutional kernels, which are essentially 2D matrices. The convolutional kernels can move with a certain stride until they completely cover the input data (27). The convolutional kernels perform convolution operations with subdatasets of the same size in the input data to extract information from the original data. To avoid high feature dimensions after convolutional operations, a pooling layer is often connected after the convolutional layer to reduce the feature dimension and computational load, thus reducing the risk of overfitting. Batch normalization layers normalize the activation values and gradient propagation in the neural network, making the network training a simpler optimization problem and accelerating the model training speed. A ReLU activation layer is added after each batch normalization layer to perform threshold operations on each element of the input. Then a dropout layer is added to prevent overfitting by setting a certain proportion of the input to *zero*. Next, a fully connected layer is added to integrate the learned features from the previous layers. Finally, a regression layer is added to calculate the mean squared error of the regression process. The structure of CNN models is shown in Figure 2A.

The data is divided into training and validation sets according to the principles of establishing an LSTM model, with every 5 numbers as one group of input, the first 149 groups as the training set, and the last 24 groups as the test set. The size of the convolutional kernel is set to a height of 3 and a width of 3, with a stride of *1* for information extraction from the input data, and is expanded according to the dimension of the input data to ensure that the convolutional kernel covers all the input data. In order to prevent information loss after the original data undergoes convolution and subsampling and to improve simulation accuracy, another convolutional layer is added after the ReLU activation layer, with the size of the convolutional kernel consistent with the first convolutional layer and twice the quantity of the first layer. Since the number of different convolutional kernels affects the model’s goodness-of-fit, different gradient numbers of convolutional kernels are set for training and prediction to determine the optimal model structure based on the RMSE value of the test set.

We used the *Adam* solver to update the network parameters by taking small steps in the direction of the negative gradient of the loss function. We set the initial learning rate to 0.005, and the number of maximum iterations to 1,000. We systematically varied the number of convolutional kernels in the first convolution layer from 4 to 150 in increments of 4 (Convolutional kernels in the second convolution layer were 8 to 300 in increments of 8), calculating the simulation performance on both the training and test sets of different models. We also mitigated the risk of overfitting by adding regularization and implementing a phased learning rate schedule, using a piecewise learn rate schedule, multiply the initial learning rate by a drop factor of 0.2 after half of the maximum epochs.

### 2.6 Constructing the NARX model

The NARX network is a powerful neural network architecture specifically designed for modeling and predicting time series data by considering both the autoregressive relationship within the time series and the influence of exogenous inputs.

The NARX network consists of two main components: the autoregressive (AR) part and the exogenous (X) part. The AR part captures the relationship between past values of the time series itself, while the X part captures the influence of the exogenous inputs on the time series. The X part can be implemented as a separate input layer or concatenated with the AR inputs. We set the outputs of the ARIMA model as the external input components of the NARX model (38).

During training, the NARX network is fed with historical data, including both the time series values and the corresponding exogenous inputs. The network learns to predict the future values of the time series based on its past values and the exogenous inputs. The training process involves adjusting the network’s weights and biases to minimize prediction errors.

The defining equation for the NARX model is


$$y\left(t\right)=f\left(\begin{array}{l} y\left(t-1\right),y\left(t-2\right),\ldots ,y\left(t-n_{y}\right),\\ u\left(t-1\right),u\left(t-2\right),\ldots ,u\left(t-n_{u}\right) \end{array}\right)$$

Where *f* represents a function that relies on the structure and connection weights of the NARX model, *y* refers to the sample data in a lagged period. *u* refers to the input series containing the time factor and the projections of the ARIMA model, y is the simulation values by the NARX model at time step *t.* Before modeling, we need to define the structure of the model. In this model, the simulated series of the ARIMA model was treated as the input, while the reported cases of HFRS were regarded as the output. Following this, the dataset was partitioned randomly into a training set (80%) for network training and a validation set (20%) for assessing the network’s generalization performance and terminating the training process to prevent overfitting (39). Since the delays of the input and the number of hidden neurons have an impact on the performance of the model, we set the delays to 5, and constructed multiple open-loop (series–parallel) architectures containing different neurons (experimented from 4 to 150 in increments of 4) for training the networks separately, using the Bayesian regularization backpropagation algorithm for updating weights during training. The predicted values from the ARIMA model were then used as inputs to the NARX model for 24-step prediction. The goodness-of-fit was calculated separately for the training and test sets, and the structure with the smallest RMSE in the test set was considered as the best-fitting NARX model structure.

### 2.7 Constructing the hybrid CNN-LSTM model

The hybrid CNN-LSTM model integrates an LSTM layer into the CNN neural network architecture. To make the input compatible with the LSTM’s dimensional requirements, a flattening layer is added before the LSTM layer. In summary, the structure of the CNN-LSTM model constructed in this study consists of the following sequence of layers: input layer, convolutional layer, pooling layer, batch normalization layer, ReLU activation layer, convolutional layer, pooling layer, batch normalization layer, ReLU activation layer, flattening layer, LSTM layer, ReLU activation layer, dropout layer, fully connected layer, and regression layer. The structure of CNN models is shown in Figure 2B. The division of the training and test sets is consistent with that of single CNN and LSTM models. We used the *Adam* solver to update the network parameters by taking small steps in the direction of the negative gradient of the loss function. We set the initial learning rate to 0.005, and the number of maximum iterations to 1,000. The number of convolutional kernels in the first convolution layer is the same as the single best-fitting CNN model, and the number of hidden units of the LSTM layer is the same as the best-fitting LSTM models. We also mitigated the risk of overfitting by adding regularization and implementing a phased learning rate schedule, using a piecewise learn rate schedule, multiply the initial learning rate by a drop factor of 0.2 after half of the maximum epochs. The model is trained using the training set data, followed by performing 24-step ahead predictions. The goodness-of-fit is calculated separately for both the training and test sets.

### 2.8 Goodness-of-fit of the ARIMA, LSTM, CNN, NARX, and CNN-LSTM models

The RMSE, MAE, and MAPE of train set and test set were calculated as indicators for evaluating the goodness-of-fit of the models mentioned above, which were given by


$$RMSE=\sqrt{\frac{1}{N}\;\overset{N}{\underset{t=1}{\sum }}\;{\left(x_{t}-y_{t}\right)}^{2}}$$

$$MAPE=\frac{100\%}{N}\;\left(\overset{N}{\underset{t=1}{\sum }}\;\frac{\left|x_{t}-y_{t}\right|}{x_{t}}\right)$$

$$MAE=\frac{1}{N}\left(\;\overset{N}{\underset{t=1}{\sum }}\;\left|x_{t}-y_{t}\right|\right)$$

Where $x_{t}$ and $y_{t}$ denote the original and simulated series, respectively.

### 2.9 Software and significant level

MATLAB 2023a (MathWorks Corporation, United States) was used to perform the models involved in the study, and Microsoft Office 2021 (Microsoft Corporation, United States) for data collection and processing. The level of significance for hypothesis testing involved in this study is set at 0.05, a two-sided *p* < 0.05 was considered statistically significant.

## 3 Results

### 3.1 Trends and seasonality of the sample data

**Figure 3:**
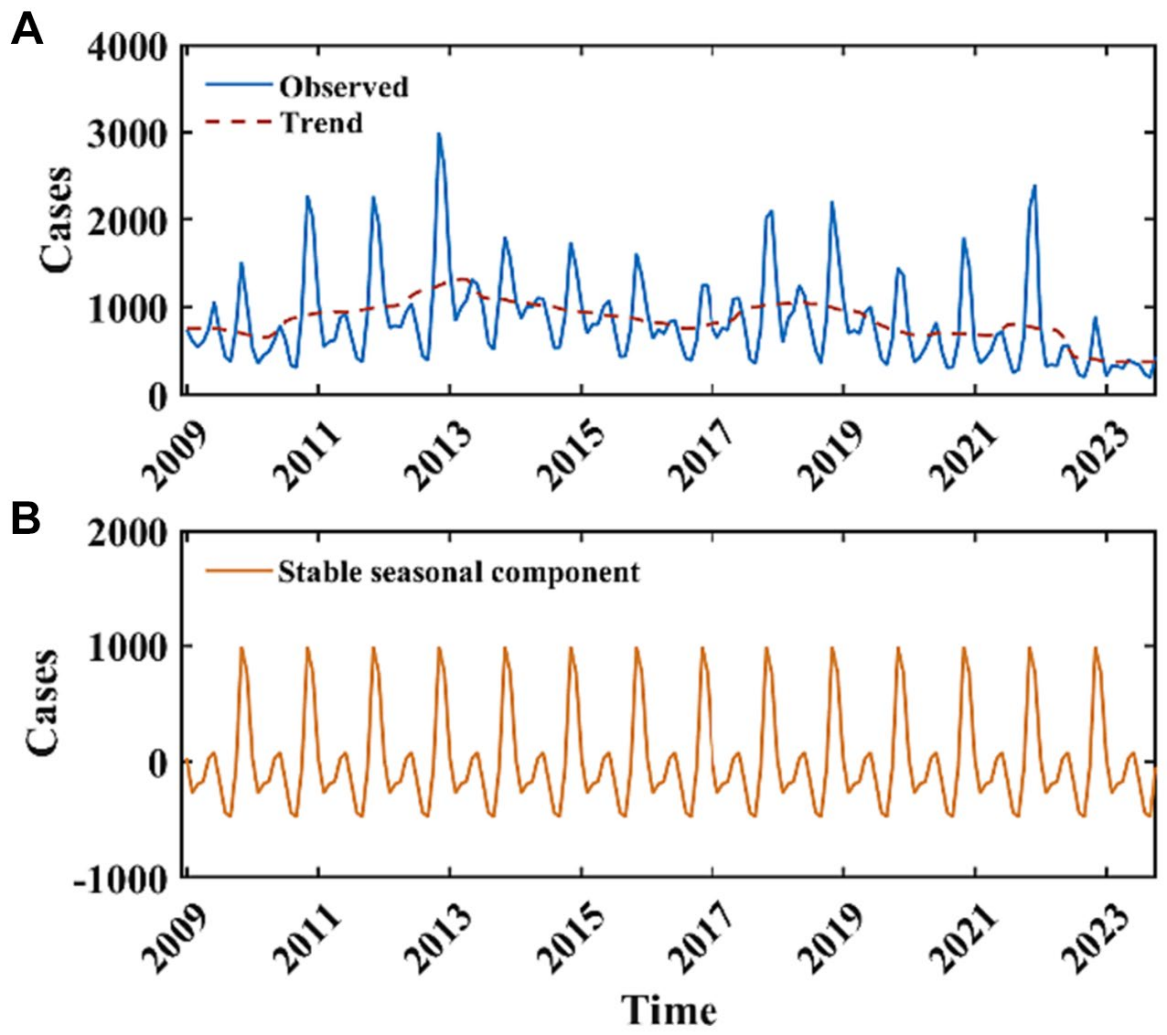
Monthly reported cases of HFRS from January 2009 to October 2023 and the decomposition of the time series data. The blue curve in **(A)** represents the monthly reported cases of HFRS, the red curve in **(A)** represents the long-term trend, **(B)** represents the stable seasonal component with a periodicity of 12 months.

Between 2009 and 2022, the average annual incidence of HFRS reported in Mainland China was 10,563 cases, with a standard deviation of 2,121. The year 2012 recorded the maximum number of cases during this interval, amounting to 13,918 instances. Annually, the case counts surpassed the 10,000 mark during the periods of 2011 to 2015 and 2017 to 2019. In the timeframe extending from January 2009 to October 2023, the mean monthly case count was 848.66 (Figure 3A). The results of the M-K test indicate that the *z*-value is −4.204, suggesting that the sample data exhibit a statistically significant decreasing trend. The results of the periodic decomposition analysis indicate that the months of November and December consistently exhibit peak incidence rates for HFRS (Figure 3B).

### 3.2 The best-fitting ARIMA model

Initially, we subjected the training dataset to the Augmented Dickey-Fuller (ADF) test, which indicated non-stationarity of the data (*t* = −1.02, *p* = 0.272). After applying first-order differencing and seasonal differencing, the data achieved stationarity (the *Ljung-Box Q-*test *χ^2^* = 89.74, *p* < 0.001), thus meeting the prerequisites for model construction. Both seasonal and non-seasonal differencing orders were identified as one. We constructed a total of 225 models by varying the combinations of model parameters and selected the optimal fitting ARIMA model based on the principle of the smallest sum of AIC and BIC values. The results indicated that the ARIMA(2,1,2)(3,1,3)_12_ model was the best-fitting ARIMA model. The model can be expressed as a polynomial of


$$\begin{array}{l} \left(1-\Phi_{1}L-\Phi_{2}L^{2}\right)\left(1-\Phi_{12}L^{12}-\Phi_{24}L^{24}-\Phi_{36}L^{36})\left(1-L\right)\left(1-L^{12}\right)\right)\\ y_{t}=\left(1+\theta_{1}L+\theta_{1}L^{2}\right)\left(1+\Theta_{12}L^{12}+\Theta_{24}L^{24}+\Theta_{36}L^{36}\right)\varepsilon_{t}. \end{array}$$

**Figure 4:**
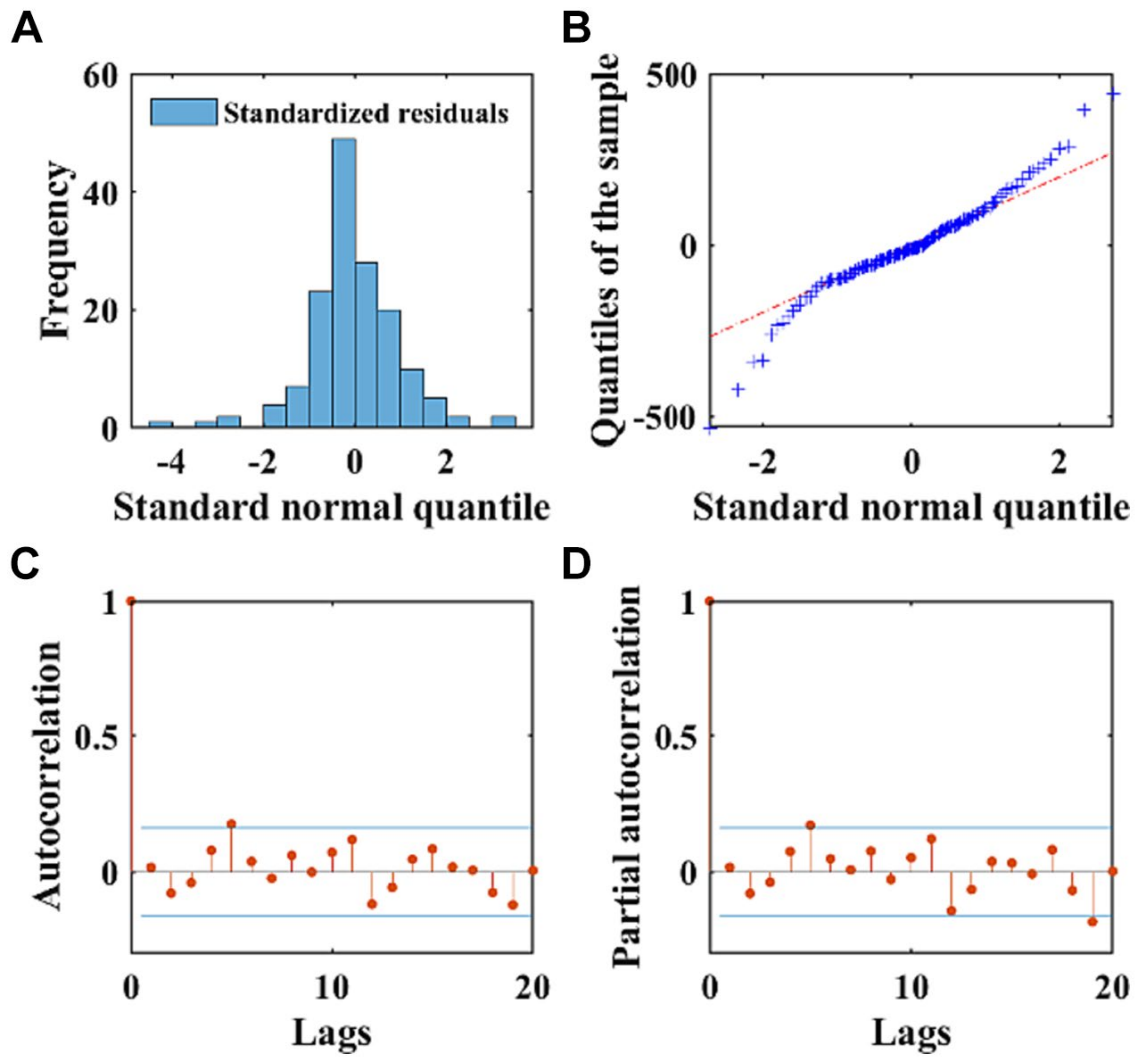
The normality and autocorrelation diagnostics of the residuals of the ARIMA model. **(A)** Displays the frequency distribution of standardized residuals using a histogram. **(B)** Exhibits the QQ plots of the residuals of the ARIMA model, with the red dashed line representing the standard normal distribution. **(C,D)** Depict the ACF and PACF of the residuals, respectively. The stem plots illustrate the values of ACF and PACF at different lags, with the blue lines indicating the ±2 times standard deviation interval.

The AIC and BIC of the ARIMA model were 1876.1, and 1908.6, respectively. We performed the autoregression and normality diagnostics on the residuals, and the result of *Ljung-Box Q*-test showed that there was no autocorrelation in the residuals (*χ^2^* = 15.34, *p* = 0.756), and the residual ACF and PACF plots showed that most of the residuals were within the ±2 times standard deviation interval (Figures 4C,D), which indicated that the fitting was successful. The histogram of the standardized residual distribution and the QQ plot (Figure 4B) of the residuals indicated that the standardized residuals showed an almost symmetrical distribution with *zero* as the boundary, and the frequency of the standardized residuals in the ±2 interval accounted for more than 80% of all (Figure 4A), which can therefore be regarded as a normal distribution. A 24-time-step prediction was performed using the ARIMA model, The fitting and predicting efficacy of the model was calculated separately, which are shown in Supplementary Table S1.

### 3.3 The best-fitting LSTM, CNN, NARX, and CNN-LSTM models

**Figure 5:**
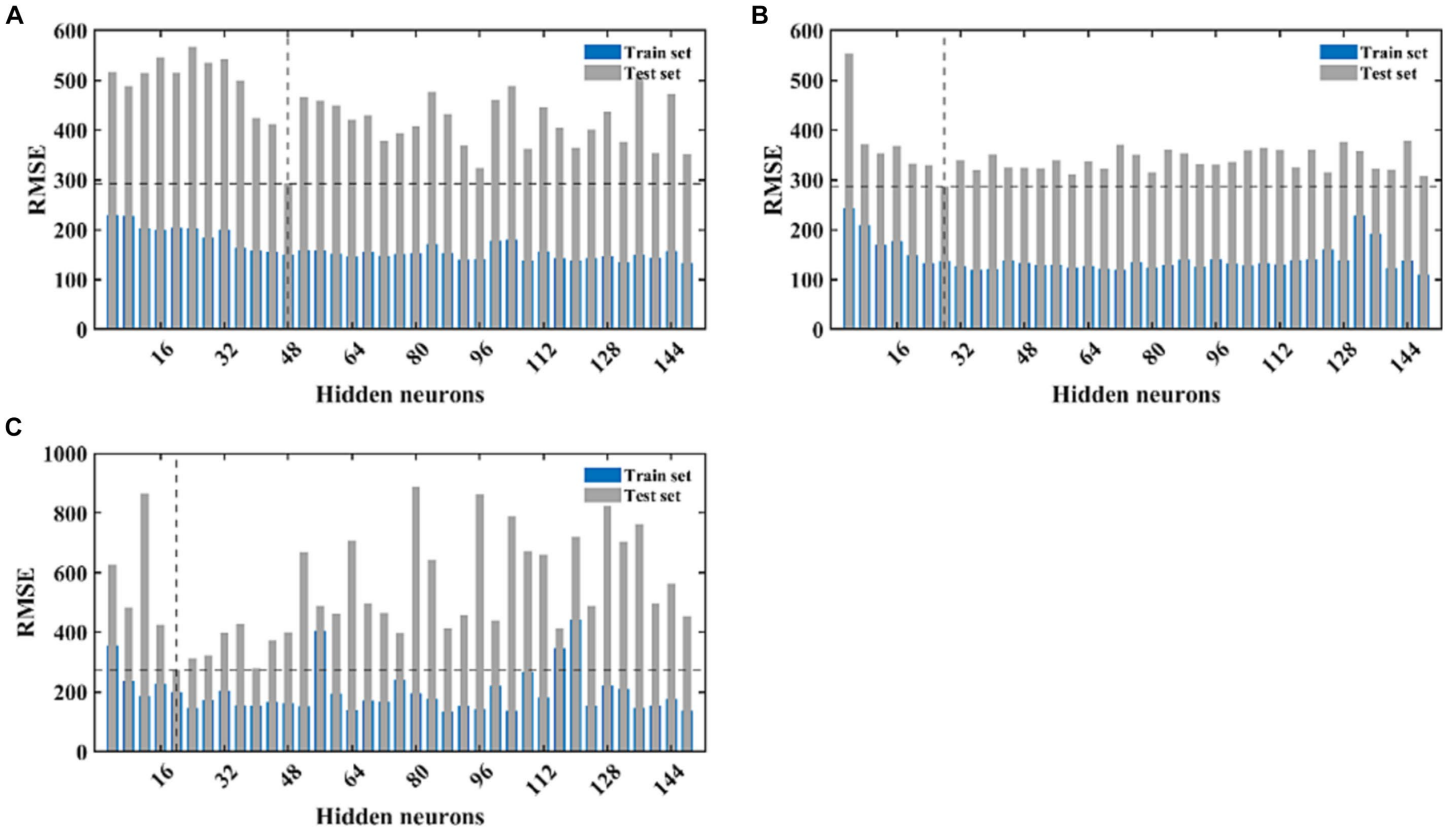
The fitting performance of LSTM, CNN, and NARX models with different neural network structures. **(A)** Represents the fitting effect of the LSTM model, **(B)** represents the fitting effect of the CNN model, and **(C)** illustrates the fitting effect of the NARX model. The blue and light green bars, respectively, represent the RMSE of the training and testing sets for each model under different structures.

The LSTM neural network model we designed consists of the input layer, LSTM layer, RELU activation layer, fully connected layer, and regression layer. This is the most basic LSTM structure. Since we pre-set the initial learning rate and maximum number of iterations, the only factor determining the quality of the LSTM model is the number of hidden neurons. As there is currently no mature method for determining the number of neurons, we constructed models with different numbers of neurons using different gradients, and calculated the goodness-of fit-of the different models using the RMSE value of the test set as the selection criterion. After continuous experimentation, we determined that the number of neurons is 48, with the smallest RMSE value on the test set. Therefore, an LSTM model with 48 hidden neurons was used for further modeling and prediction (Figure 5A). Using this model, we made 24-step backward predictions and calculated the goodness-of-fit indicators for both the training and test sets.

The complexity of the network structure, the number and size of the convolutional kernels, and the initial learning rate of the CNN model may all have an impact on the model’s fitting performance. Setting different gradients for each variable will increase the time cost of modeling. Therefore, we fix the values of most parameters and only set different gradients for the number of convolutional kernels. Since our dataset takes every 5 values as an input and the next value as an output, we set the size of the convolutional kernel to 3×3, moving with a stride of 1, and expanding outward to the same size as the input data. The model parameters will increase with the increase in the number of convolutional kernels, so the number of convolutional kernels should not be set too high. We set the gradient to 4, increasing from 4 to 150. In the second convolutional layer of the model, the number is set to twice that of the first layer. We calculate the fitting performance of these 30 models separately and make predictions on the training set data. The model with the smallest training set RMSE is selected as the best-fitting CNN model. After calculation, the model performs best on the test set when the number of convolutional kernels in the first layer is 28 and in the second layer is 56 (Figure 5B). We retrained and predicted using the model, and separately calculated the fitting performance on the training and test sets.

The NARX neural network is a method specifically used to construct time series models and requires external input data. Therefore, we used the output of the ARIMA model as the input for the NARX. We first determined a lag order of 5 and then calculated the model’s performance under different numbers of hidden neurons. Similarly, we selected the model with the smallest test set RMSE value as the best NARX model. After calculation, the model’s predictive performance was best when the number of hidden neurons was 20 (Figure 5C). We continued to retrain and predict using this model and separately calculated the fitting performance on the training and test sets. The training progress of the NARX model, target-output time series results, regression results of the training and test sets, and residual autocorrelation diagnostic results are shown in Supplementary Figures S1–S4.

We combined the trained CNN and LSTM models to obtain the optimally fitted CNN-LSTM model. This model was utilized to perform 24-step ahead forecasting, and the fitting performance for both the training and test sets was calculated.

### 3.4 Comparison of the simulation and prediction effects of the ARIMA, LSTM, CNN, NARX, and CNN-LSTM models

**Figure 6:**
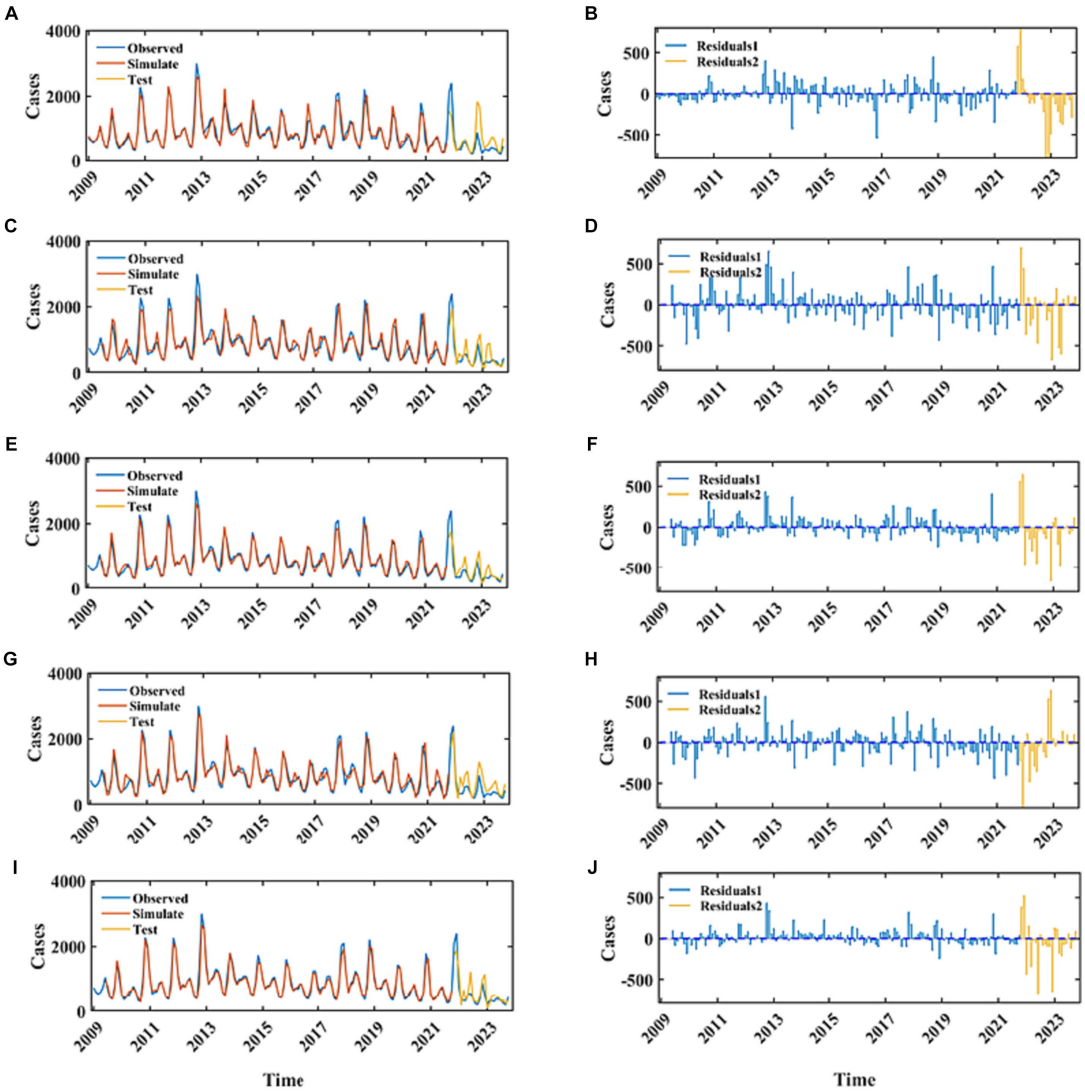
The fitting and prediction performance of the best-fitted ARIMA, LSTM, CNN, NARX, and CNN-LSTM models. **(A,C,E,G,I)** Represent the fitting effect between the simulated values and the observations for the ARIMA, LSTM, CNN, NARX, and CNN-LSTM models. The blue curve represents the actual incidence of HFRS, the red curve represents the fitted values for the training set, and the yellow curve represents the predicted values for the testing set. **(B,D,F,H,J)** Represent the residuals of these five models. The blue stem plot indicates the residuals of the training set, and the yellow stem plot represents the residuals of the testing set.

Employing the ARIMA, LATM, CNN, NARX, and CNN-LSTM architectures, a high degree of fidelity in the fitting of train datasets was achieved (Figure 6). Based on the comprehensive RMSE, MAPE, and MAE indicators from the train set, the superior model fitting hierarchy is as follows: CNN-LSTM, CNN, ARIMA, NARX, and LSTM. The CNN-LSTM model, which demonstrated the best fitting performance, had RMSE, MAPE, and MAE values of 93.77, 7.59%, and 64.37, respectively. Compared to the ARIMA model, these values represent a reduction of 28.63, 30.68, and 31.91%, respectively. Relative to the LSTM model, the reductions are 48.44, 49.64, and 50.71%, respectively. When contrasted with the CNN model, the reductions are 20.43, 27.51, and 25.94%, respectively. Finally, in comparison to the NARX model, the reductions are 36.79, 47.40, and 41.98%, respectively. Subsequent to a rigorous evaluation of the test set based on these performance indices, the CNN-LSTM and NARX models were found to have a commensurable predictive capability, marginally superior to that of the CNN and LSTM frameworks. In stark contrast, the ARIMA model’s predictive capacity was significantly inferior. Quantitatively, the ARIMA model’s RMSE on the testset exhibited increments of 27.85, 32.63, 33.12, and 36.59% relative to the LSTM, CNN, NARX, and CNN-LSTM models, respectively. Concurrently, the model’s MAE values were augmented by 17.13, 26.89, 36.32, and 26.34%, and the MAPE values were escalated by 21.94, 28.50, 33.30, 30.84%, correspondingly. When juxtaposed with the hybrid CNN-LSTM construct, the RMSE values for the single CNN and LSTM models on the test set were found to be elevated by 5.88 and 12.11%, respectively. Delving into the comparative predictive efficacy of the NARX and CNN-LSTM models, the NARX model’s RMSE value was observed to be 5.19% higher, albeit with a decrement of 15.68% in MAPE and a 3.7% reduction in MAE (Table 1).

**Table 1:** Comparison of the simulation and prediction effects of the ARIMA, LSTM, CNN, NARX, and CNN-LSTM models.

Models	Simulating power			Predicting power		
	RMSE	MAPE	MAE	RMSE	MAPE	MAE
ARIMA	131.38	10.95%	94.54	426.83	52.89%	274.32
LSTM	181.87	15.07%	130.58	307.94	43.83%	214.13
CNN	117.85	10.47%	86.91	287.56	38.67%	196.14
NARX	148.35	14.43%	110.95	285.48	33.68%	182.97
CNN-LSTM	93.77	7.59%	64.37	270.66	38.96%	189.73
ARIMA vs. LSTM	−38.43%	−37.63%	−38.12%	27.85%	17.13%	21.94%
ARIMA vs. CNN	10.30%	4.38%	8.07%	32.63%	26.89%	28.50%
ARIMA vs. NARX	−12.92%	−31.78%	−17.35%	33.12%	36.32%	33.30%
ARIMA vs. CNN-LSTM	28.63%	30.68%	31.91%	36.59%	26.34%	30.84%
LSTM vs. CNN	35.20%	30.52%	33.44%	6.62%	11.77%	8.40%
LSTM vs. NARX	18.43%	4.25%	15.04%	7.29%	23.16%	14.55%
LSTM vs. CNN-LSTM	48.44%	49.64%	50.71%	12.11%	11.11%	11.40%
CNN vs. NARX	−25.88%	−37.82%	−27.65%	0.72%	12.90%	6.72%
CNN vs. CNN-LSTM	20.43%	27.51%	25.94%	5.88%	−0.75%	3.27%
NARX vs. CNN-LSTM	36.79%	47.40%	41.98%	5.19%	−15.68%	−3.70%

## 4 Discussion

In recent years, the incidence of HFRS in the mainland China has maintained a relatively stable level. Time series data are comparatively accessible, as well as amenable to computation and analysis. Consequently, time series analysis offers economic benefits and saves on time costs. Analyzing the epidemiological trends of diseases is advantageous for the government to formulate intervention measures in advance and to allocate disease prevention and control resources rationally. The temporal epidemiological analysis of HFRS in the mainland of China spanning from January 2009 to October 2023 exhibits a decrement trend. The morbidity rates of HFRS are characterized by conspicuous seasonality, providing empirical substantiation for the deployment of an ARIMA modeling framework. Our analysis of the seasonal pattern of HFRS indicates that the peak incidence of HFRS occurs in November and December each year, which is consistent with the findings of Lv et al. (32). A pivotal aspect of ARIMA model construction involves the ascertainment of the differencing and autoregressive moving average parameters, denoted as *p, d, q,* along with their seasonal counterparts *P*, *D*, *Q*. Subsequent to order specification, parameter estimation is undertaken, necessitating the generation and scrutiny of the ACF and PACF plots derived from the differenced time series data. The determination of model order is predicated upon the inspection of the decay patterns within these correlograms, which, albeit informative, introduces a modicum of subjectivity into the model selection process. To mitigate this, the optimal ARIMA model is adjudicated based on the minimization of the combined AIC and BIC. Residual diagnostics of the optimal ARIMA model reveal that the residuals approximate a Gaussian distribution, evidencing the absence of autocorrelation and thus, endorsing the adequacy of the model fit. Notwithstanding, the presence of residual autocorrelation exceeding the threshold of twice the standard deviation at lag 5 intimates the potential perturbation induced by anomalous observations. The congruence between the fitted ARIMA model and the empirical data is substantiated by the proximity of MAE and RMSE metrics to the value of 100, reflecting a robust predictive performance of the model. Despite a comparative diminution in predictive accuracy on the validation set vis-à-vis the training cohort, a convergence in the trend trajectories between forecasted and observed values is discernible, underpinning the model’s utility in prognosticating pivotal inflection points in HFRS incidence. However, the model’s predictive divergence is amplified for the Nov and Dec 2022 and Jan 2023 data points, which may be attributable to the inherent elevation of cases during these intervals historically. This discrepancy underscores the challenges in forecasting when seasonal peaks are pronounced, even when utilizing a model that accommodates seasonality by referencing temporally analogous historical incidence data.

Owing to the suboptimal performance of the ARIMA model in capturing the nonlinear dynamics within time series data (40), we aim to ameliorate this limitation via the implementation of neural network architectures. Conventional RNNs are plagued by the issue of gradient vanishing, wherein the backpropagation of error gradients becomes ineffectual over extended temporal sequences, disproportionately magnifying the influence of proximal inputs on the predictive model. LSTM networks, however, surmount this challenge through an intricate gating mechanism, equipped with a dedicated Cell state for the retention of protracted temporal dependencies, which is conducive to enhancing both the fidelity of model fit and prognostic precision. Empirical evidence indicates that the LSTM framework exhibits superior proficiency in fitting the empirical data and surpasses the ARIMA model in predictive performance on the validation set. The LSTM paradigm not only forecasts the directional momentum of future data points but also demonstrates a reduced goodness-of-fit metric on the test set relative to the ARIMA. Nonetheless, the LSTM’s fit on the training dataset is somewhat inferior compared to the ARIMA model, potentially attributable to the LSTM’s less robust encapsulation of the seasonal fluctuations inherent in the time series (41).

The CNN represents an intricate neural network topology that leverages convolutional filters to perform feature extraction from raw data. This is achieved through convolutional operations that integrate kernel functions with input data, subsequently followed by subsampling techniques aimed at dimensionality reduction of the convolved features. This process iterates through additional layers of convolution and subsampling before reaching a series of fully connected layers that perform an integration of learned weights. Distinct from the LSTM network, which prioritize temporal recursion and sequence modeling, CNNs are tailored towards the extraction and high-level abstraction of spatial and temporal features from input data. Neurons in the convolutional layers are connected to subregions of preceding layers rather than being fully connected, as is the case in other types of neural networks. Neurons exhibit no response to areas outside of these subregions within the sample, and these subregions may overlap, leading to spatially correlated outcomes produced by the neurons of a CNN. CNNs can reduce the number of parameters by reducing the number of connections, sharing weights, and employing subsampling techniques. This demonstrates excellent performance in low-level feature extraction and feature representation. Empirical evaluations indicate that CNNs possess superior emulation fidelity and predictive capabilities. Relative to ARIMA and LSTM approaches, CNNs have demonstrated a heightened ability to anticipate pivotal shifts in future data trajectories with a diminished incidence of outliers. This enhanced predictive capacity may stem from the CNN’s iterative convolutional engagement with the raw data during the training phase, ensuring a more exhaustive exploitation of the underlying informational content. We also mitigated the risk of overfitting by adding regularization, adding the dropout layer, and implementing a phased learning rate schedule.

In this study, the fitting results of the ARIMA model are utilized as external input, and this modeling approach can be considered as a hybrid modeling method (42). Our proposed combined approach, which integrates the linear ARIMA method and the nonlinear ANNs technique, aims to uncover various types of relationships within disease series characterized by distinct periodicity and seasonal variation, thereby enhancing prediction capability. In the context of time series modeling, the inherent periodicity within the data is a crucial reference factor for predicting disease trends. The NARX model combines the strengths of the ARIMA model and the NARX model, addressing their respective proficiency domains, namely the linear and nonlinear components. By assessing the correlation between the ARIMA method’s forecasted outcomes and the observed values, it becomes possible to extract residual clues from the data. In the hybrid NARX approach, the time variables and values simulated and forecasted by the ARIMA method are considered as input variables, with the actual data serving as the target values to be predicted, capturing both linear and nonlinear components simultaneously (38). This hybrid methodology demonstrates enhanced predictive capabilities and merits broader dissemination. The performance analysis reveals that the NARX model exhibits inferior performance on the training set compared to the ARIMA and CNN models, but outperforms the LSTM model. Moreover, the predictive efficiency of the NARX model surpasses that of the ARIMA, LSTM, and CNN models.

The results show that the CNN-LSTM model has better simulation performance for the training data than other models. For the test set predictions, the CNN-LSTM model has a fitting goodness similar to the NARX model, with RMSE values lower than NARX, and MAPE and MAE higher than those of the NARX model. The simulation results of the five models show that the hybrid models have better fitting and predictive efficiency than a single time series model.

The development of any time series model involves the identification and extraction of features from existing data, which are then utilized to simulate unknown scenarios. The model’s performance in fitting the training set reflects its ability to capture the original data, while its predictive performance on the test set demonstrates its generalization ability. Prior to establishing the ARIMA model, seasonal decomposition was conducted to specify the periodicity of the time series, enhancing the model’s fitting performance. However, in the establishment of other neural network models, no prior information was provided, necessitating the learning and presentation of long-term trends, seasonality, and random fluctuations in the fitting results. The ARIMA model outperformed the NARX and LSTM models in fitting the training set, but its predictive performance on real sample data, which contains both regular and random components, was inferior to that of the neural network models. The CNN model performs feature extraction from raw data through convolution with kernels, followed by dimension reduction through pooling, leading to a partial loss of original data information, but still exhibits outstanding fitting capabilities. Additionally, the wider applicability of the CNN model allows it to handle not only time series data but also higher-dimensional data. However, As the CNN model is essentially a feed-forward neural network, it may encounter issues of gradient vanishing or exploding during training, potentially leading to weaker long-term predictive performance compared to LSTM and NARX. At least, with a test set sample size of 12, the CNN is speculated to exhibit performance similar to RNNs in short-term prediction. The CNN-LSTM model effectively combines the information extraction capability of the CNN model with the recurrent prediction ability of LSTM, demonstrating excellent simulation performance in both the training and test sets. This suggests the feasibility of hybrid models, even in cases where the original data lacks periodicity, and indicates the potential for using this model for predicting time series data of other diseases. Furthermore, the CNN-LSTM model exhibits better robustness than NARX, as NARX did not exhibit the best fitting performance in the training set, yet performed well in the test set. Therefore, when researchers lack real data as a basis for selecting model parameters, the CNN-LSTM model is recommended as a more robust approach for predicting unknown data.

## 5 Limitations

Certainly, this study has some limitations. Firstly, although the sample data were rigorously reported by various levels of health institutions, there may still be potential reporting bias, especially during January–February 2020 when China was experiencing the initial phase of the COVID-19 epidemic, and the country was under lockdown, which might have limited case diagnoses. Secondly, for the neural network models involved in this study, there is currently no mature method to determine the most appropriate number of hidden neurons. Identifying the best model requires continuous trial and parameter adjustment. Thirdly, the models involved in this study are all data-driven, requiring a large amount of historical data for learning and training, and can only make short-term predictions. If the values and quantity of the sample data change, the best time series models in this study may not necessarily be applicable.

## 6 Conclusion

During the period from January 2009 to October 2023, the incidence of HFRS across mainland China have been on a general decline, with seasonal peaks consistently recorded in the months of November and December. The hybrid CNN-LSTM model, which combines the data extraction capabilities of CNN with the recurrent recursive abilities of LSTM networks, exhibits commendable fitting adequacy and robustness on both train and test datasets. This CNN-LSTM model stands out due to its flexibility, as it imposes no prerequisites on the underlying distribution of the data, and its theoretical proficiency in adeptly fitting higher-dimensional data sets is noteworthy. Therefore, it is advised that entities involved in public health policy formulation consider the adoption of the CNN-LSTM methodology as a pivotal instrument for the analysis and projection of disease.

## Data availability statement

The original contributions presented in the study are included in the article/Supplementary material, further inquiries can be directed to the corresponding authors.

## Ethics statement

Ethical approval was not required for the study involving humans in accordance with the local legislation and institutional requirements. Written informed consent to participate in this study was not required from the participants or the participants' legal guardians/next of kin in accordance with the national legislation and the institutional requirements.

## Author contributions

ZW: Data curation, Formal analysis, Investigation, Methodology, Software, Visualization, Writing – original draft, Writing – review & editing. CY: Conceptualization, Data curation, Formal analysis, Validation, Writing – review & editing. BL: Investigation, Project administration, Validation, Visualization, Writing – review & editing. HW: Methodology, Software, Validation, Writing – review & editing. ZX: Resources, Supervision, Validation, Writing – review & editing. ZF: Project administration, Resources, Supervision, Validation, Writing – review & editing.

## Footnotes

^1^ http://www.nhc.gov.cn/jkj/new_index.shtml
