# Supplementary material for: Comparison of simulation and predictive efficacy for hemorrhagic fever with renal syndrome incidence in mainland China based on five time series models
Source: Front Public Health. 2024 Feb 29;12:1365942. doi: 10.3389/fpubh.2024.1365942 (PMC10941340; doi:10.3389/fpubh.2024.1365942)
Supplement: Supplementary file 3 [file Data_Sheet_1.docx]

**Supplementary Materials**


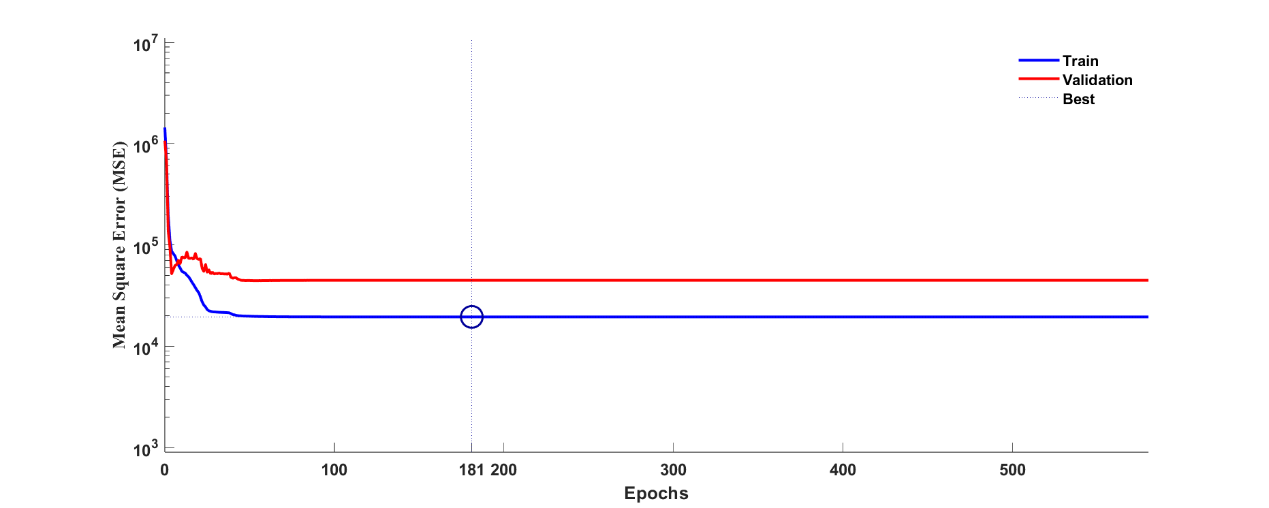


**Figure S1 The training progress of the NARX model** The blue and red curves represent the change in Mean Squared Error (MSE) for the training and validation datasets. The MSE values for train and validation sets cease to decrease after 181 iterations, leading to the termination of the model's training process.


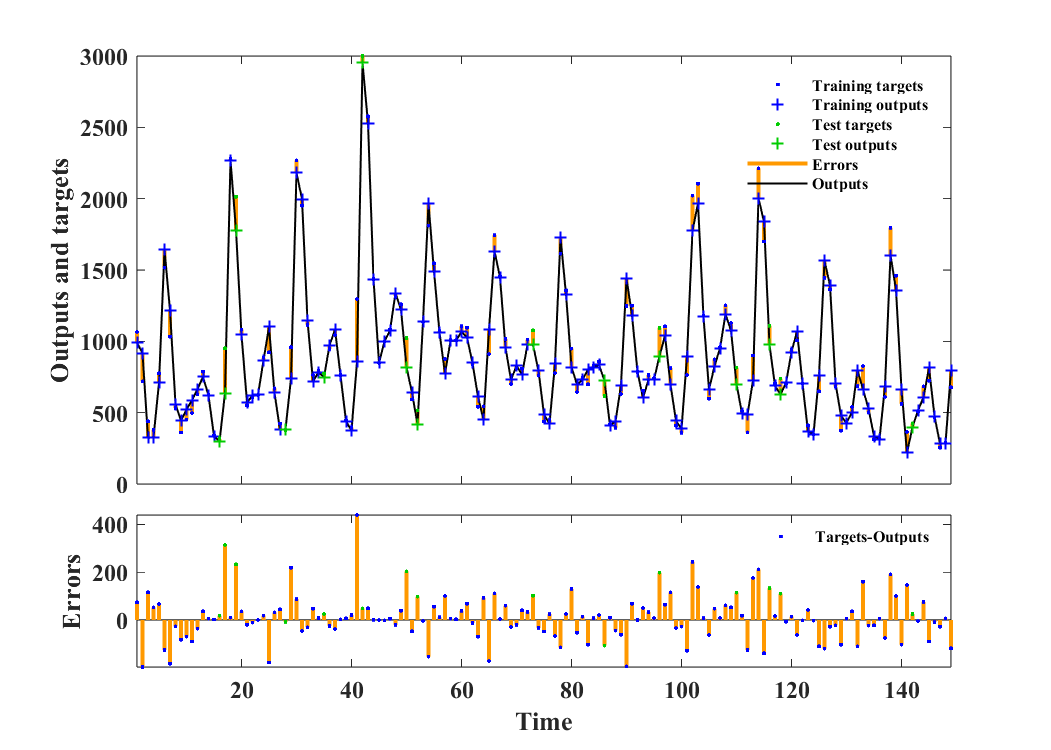
 **Figure S2 The target-output temporal response of the NARX model** The red curve denoting the model's outputs. The blue and yellow dots represent the training target values and testing set, respectively, while the cross symbols signify the model fitting results for the training and testing sets. The yellow line segment represents the error values.


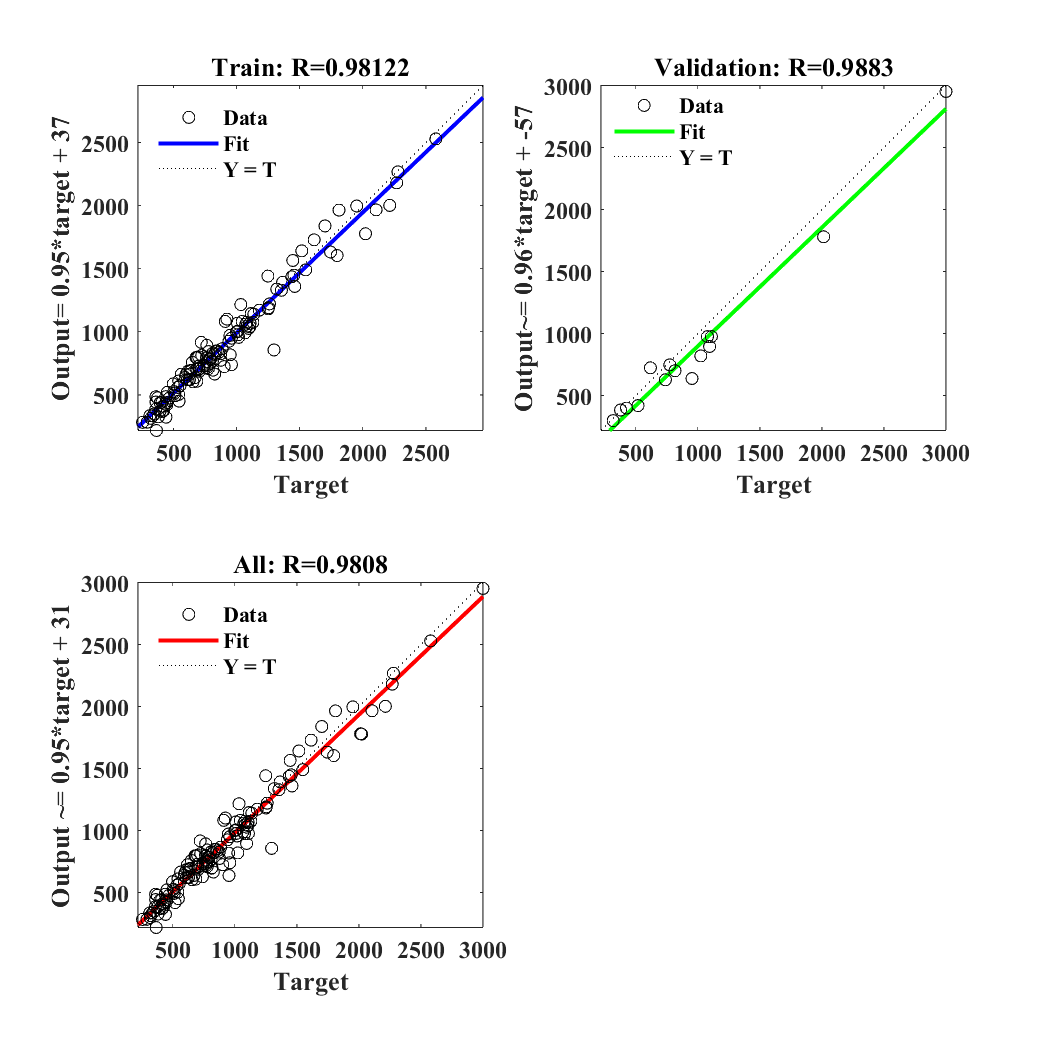


**a**

**b**

**c**

**Figure S3 Regression analysis on various datasets of the NARX model**  The hollow circles represent the model's fitted data. Panels a, b, and c respectively denote the regression results for the training set, testing set, and the entire dataset. The R-squared values for all three datasets exceed 98%, substantiating the robustness of the model's fitting.


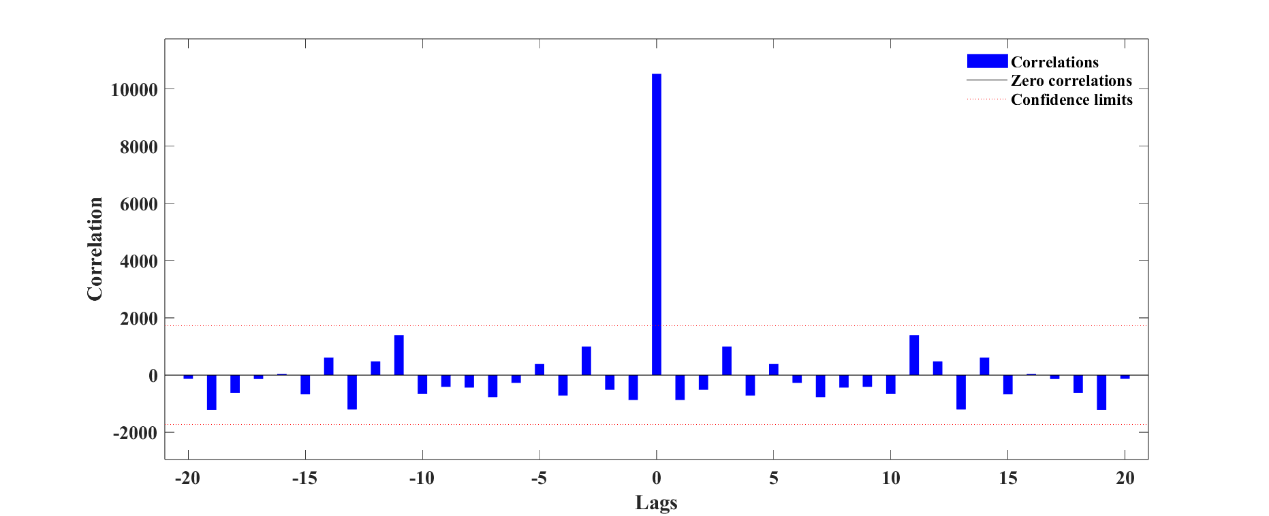
 **Figure S4 Autoregressive tests on the residuals of the NARX model** The blue bars represent the autoregressive results of the NARX residuals, all of which fall within the confidence interval, confirming the well-fitted nature of the model.
